# TiZrHf-Based B2-B19′/B19-High Entropy Shape Memory Alloys: A Review and Recent Advances

**DOI:** 10.3390/ma19143064

**Published:** 2026-07-16

**Authors:** Yoko Yamabe-Mitarai

**Affiliations:** Graduate School of Frontier Science, The University of Tokyo, Chiba 277-8561, Japan; mitarai.yoko@edu.k.u-tokyo.ac.jp

**Keywords:** high-entropy alloys, shape memory alloy, martensitic transformation, B2, B19, B19′, shape memory effect, superelasticity, erastocaloric effect

## Abstract

**Highlights:**

- High entropy shape memory alloys with B2-B19′ or B2-B19 transformation are reviewed.
- Martensitic transformation temperature, shape memory effect, and superelasticity are summarized.
- Machine learning for predicting martensitic transformation temperature and thermal hysteresis is reviewed.

**Abstract:**

This review summarizes the development of Ti-based high-entropy and multi-principal element shape memory alloys (SMAs), with a particular focus on TiZrHfCoNiCu, TiHf(Zr)Ni(Pt)Pt, and TiPd-based systems. Alloy composition and heat treatment significantly influence martensitic transformation temperatures (MTTs), thermal hysteresis, superelasticity (SE), shape memory effect (SME), and elastocaloric effect (eCE) through precipitation reactions, compositional partitioning, and lattice strain effects. These parameters are summarized in the tables. Furthermore, recent advances in machine learning have provided powerful tools for predicting MTTs and thermal hysteresis. Important features governing phase transformation behavior, as well as suitable regression models for predicting MTT and thermal hysteresis, are introduced. These developments demonstrate a transition from empirical alloy development toward data-driven and physics-informed design of next-generation HE-SMAs.

## 1. Introduction

Shape memory alloys (SMAs) exhibit unique properties, including the shape memory effect (SME) and superelasticity (SE), in response to changes in temperature and stress. The SME originates from the reverse martensitic transformation (MT) from martensite to austenite upon heating, whereas SE results from stress-induced martensitic transformation and its subsequent reverse transformation during unloading. The operating temperature of SMAs is determined by the martensitic transformation temperature (MTT), defined by four characteristic temperatures: martensite start (*M*_s_), martensite finish (*M*_f_), austenite start (*A*_s_), and austenite finish (*A*_f_) temperatures.

Among various SMAs, TiNi-based alloys are the most extensively studied and commercially utilized because of their large recovery strain (8~10%), excellent SE, superior fatigue resistance, and outstanding biocompatibility. Consequently, they have been widely applied in actuators, biomedical devices, and aerospace components [1]. In binary TiNi alloys, the MT occurs from the B2 austenite phase to the B19′ martensite phase. However, the MTT is generally below 343 K and is highly sensitive to alloy composition, particularly the Ni concentration. The production cost of TiNi remains relatively high because Ti is highly susceptible to oxidation, and the alloys exhibit poor workability.

To overcome these limitations, Cu-based SMAs, including Cu-Al, Cu-Zn, and Cu-Sn, have been extensively investigated [2]. Compared with TiNi alloys, Cu-based SMAs offer low material cost, higher electrical and thermal conductivity, and superior cold workability [3]. Their crystal structures strongly depend on alloy composition. The austenite phase generally exhibits B2, DO3, L2_1_ (Heusler) structures, whereas the martensite phase adopts 2H, 9R, and 18R structures. Among them, Cu-Al-Mn alloys with the Heusler structure exhibit SE comparable to that of TiNi and have been successfully commercialized for biomedical applications such as ingrown toenails [3].

Fe-based SMAs have also attracted considerable attention because of their low product cost, superior workability and weldability, stable SE, and relatively low temperature dependence of the critical stress for stress-induced martensitic transformation [4]. Depending on alloy composition, the M occurs from γ-fcc austenite to either ε-hcp martensite or α-bcc (α′-bct) martensite. Owing to their excellent mechanical properties and low cost, Fe-based SMAs are promising materials for large-scale civil engineering structures, vibration-damping systems, sensing devices, tube couplings, and reinforced concrete applications [4].

The SMAs described above are thermally activated; therefore, their response speed is inherently limited by heat transfer and is typically below 10 Hz [3]. To achieve faster actuation, magnetic-field-induced SMAs have been developed [3]. Ni_2_MnGa, which possesses a Heusler-type austenite structure, exhibits a giant magnetic-field-induced recoverable strain exceeding 10%. However, its practical application has been hindered by intrinsic brittleness and low output stress [5]. The martensite structure is highly composition-dependent and includes 5M, 7M, 6M, 10M, and 14M modulated structures. More recently, NiCoMnIn alloys have been developed to overcome the brittleness and low output stress associated with Ni_2_MnGa [6]. The magnetic SMAs are candidates for high-speed actuators.

Despite these advances, the MTTs of most commercially available SMAs remain below 473 K, although some Fe-based SMAs exhibit MTT approaching 573 K. To extend the operating temperature range, extensive efforts have also been devoted to developing high-temperature shape memory alloys (HTSMAs) by alloying TiNi with elements such as Zr, Hf, Pd, and Pt. Although these additions effectively increase the MTT, plastic deformation becomes increasingly severe at high temperatures, limiting functional stability and practical applicability [7,8].

To overcome these limitations, high-entropy alloys (HEAs) and medium-entropy alloys (MEAs) have recently attracted considerable attention as a new class of SMAs. These alloys consist of multiple principal elements in equiatomic or near-equiatomic concentrations and are expected to exhibit enhanced strength due to severe lattice distortion and sluggish diffusion effects [9,10]. In general, increasing configurational entropy, $∆S_{mix}=-R\sum_{i=1}^{n}C_{i}lnC_{i}$, stabilizes the disordered solid-solution phase, which would appear unfavorable for the formation of ordered phases such as the B2 phase required for shape memory behavior. Here, R is the gas constant and $C_{i}$ is the atomic percentage of the ith element. Nevertheless, Sheng et al. demonstrated the stability of a high-entropy B2 phase [11], and Firstov et al. successfully introduced the HEA concept into SMAs by developing the first high-entropy shape memory alloys (HESMAs) exhibiting martensitic transformation and SME [12,13]. The principles for selecting alloying elements in multi-principal element SMAs have been summarized in [14]. Substitutional elements are generally chosen based on their high mutual solubility with the principal elements and similar electronic configurations, thereby maintaining phase stability while enabling systematic modification of transformation behavior and mechanical properties. According to this classification, alloying elements can be divided into five groups: Ti-equivalent (Ti, Zr, and Hf), Nb-equivalent (Nb and Ta), V-equivalent (V, Cr, Mo, and W), Ni-equivalent (Ni, Pd, Pt, Cu, Au, Mn, Fe, and Co), and Al-equivalent (Al, Ga, In, and Zn).

Since the pioneering work of Firstov, extensive research has been devoted to HESMAs. Among the various alloy systems, TiZrHf-based HESEA have attracted particular attention because two MT exhibits such as the characteristics of conventional Ti-based SMAs exhibiting the β-α″ martensitic transformation and TiNi(Pd)-based SMAs exhibiting B2 → B19′ (or B2 → B19) transformation. Several TiZrHf-based HESEA exhibiting β-α″ transformation, such as TiZrHfAlNb [15], TiZrHfNbTaSn [16], TiZrHfNbTaAl [17], TiZrHfNbTa [14], and TiZrHfNbSn [18] have been reported.

This review specifically focuses on TiZrHf-based HESMAs exhibiting the B2 → B19′ or B2 → B19 MT. The effects of alloy composition, heat treatment, microstructural evolution, martensitic transformation behavior, and functional properties are systematically summarized. In addition, recent advances in machine learning-assisted alloy design for TiZrHf-based HESMAs are reviewed to highlight emerging strategies for accelerating alloy development.

Besides TiZrHf-based HESMAs, other HESMA systems have also been explored, including Fe-based HESMAs exhibiting γ → ε and with γ → α (or α′) MTs [19,20] and NiMnGaCoGd high-entropy ferromagnetic SMAs [21,22]. A comprehensive review covering these broader classes of HESMAs has been published elsewhere [23]. Therefore, these alloy systems are not discussed further in the present review.

## 2. TiZrHfNiCoCu: B2-B19′

The first HESMA exhibiting B2-B19′ MT was reported by Firstov et al. [12,13] in TiZrHfNiCoCu alloys. Their alloy design was based on the valence electron concentration (VEC), as structural stability in transition-metal alloys correlates with VEC. Previous studies suggested that the B2 phase is stabilized within a narrow VEC range centered around 7 e/a [11], which corresponds to the conventional TiNi SMAs. Based on this concept, Firstov et al. designed equiatomic Ti_16.67_Zr_16.67_Hf_16.67_Co_16.67_Ni_16.67_Cu_16.67_, with a VEC of 7 and a high mixing entropy. The alloy composition was optimized by maintaining the equiatomic composition of Ti, Zr, and Hf while varying the Co, Ni, and Cu contents under the A_50_B_50_ stoichiometry A = (Ti, Zr, Hf) and B = (Co, Ni, Cu). Alloys containing Co exhibited a B2 structure at room temperature, whereas Ti_16.67_Zr_16.67_Hf_16.67_Ni_25_Cu_25_ indicated a B19′ structure. The corresponding MTTs are summarized in Table 1. Bending test demonstrated complete shape recovery, confirming the shape memory capability of these alloys. In a subsequent study, the effect of Co addition was systematically investigated [13]. Reducing the Co content from 16.67 at% to 10 or 5 at% promoted MT, revealing that Co acts as a strong B2 stabilizer. In particular, the substitution of Ni by Co markedly suppressed the MT. Among the investigated compositions, Ti_16.67_Zr_16.67_Hf_16.67_Co_10_Ni_25_Cu_15_ exhibited the most attractive combination of properties, including narrow thermal hysteresis and the largest transformation strain. These characteristics were attributed to the improved crystallographic compatibility and reduced volume change between the austenite and martensite phases.

Following the pioneering work of Firstov et al., extensive studies have been conducted on the TiZrHfCoNiCu to clarify the effects of heat treatment and composition on MT, SME, and SE [24,25]. Initial investigations focused on Ti_16.67_Zr_16.67_Hf_16.67_Co_10_Ni_25_Cu_15_. The influence of solution treatment temperature was systematically examined [25]. As summarized in Table 1, solution treatment at 1273 K resulted in lower MTTs than those of the as-cast alloys reported by Firstov et al. [12,13]. Further reductions in MTT were observed after solution treatment at 1173 and 1223 K, followed by furnace cooling (FC). These changes were associated with variations in the volume fraction of Ti_2_Ni precipitates. A higher fraction of Ti_2_Ni was observed in FC samples than in water-quenched (WQ) samples, leading to Ti depletion in the matrix and consequently lower MTTs. Consistent with this microstructural evolution, the WQ samples exhibited superior SME compared with the FC samples, whereas the SE response was largely unaffected by the cooling condition, as summarized in Table 2.

The effects of aging treatment on Ti_16.67_Zr_16.67_Hf_16.67_Co_10_Ni_25_Cu_15_ were subsequently investigated [26]. Both solution-treated and aged samples exhibited excellent SE behavior, with nearly complete strain recovery at room temperature. Notably, perfect SE was retained at temperatures up to 75 K above *A*_f_ (373 K), significantly exceeding the typical temperature range of approximately 30 K above *A*_f_ observed in conventional TiNi alloys. This exceptional behavior highlights the enhanced stability of stress-induced martensitic transformation in this alloy system.

The compositional dependence of MT was investigated in the Ti_50-2x_Zr_x_Hf_x_Ni_50-2x_Cu_x_Co_x_ system (x = 1, 5, 10, and 17 at%) [27]. Clear MT was observed only for x = 1. Although X-ray diffraction (XRD) confirmed a B2 structure for x = 5, differential scanning calorimetry (DSC) revealed weak and broad transformation peaks. For x ≥ 10, MT disappeared completely, accompanied by the formation of Ti_2_Ni and Ni_4_Ti_3_ precipitates. These results suggest that increasing compositional complexity and mixing entropy suppresses MT.

A more detailed compositional study was subsequently performed by varying the concentration of Ni-group elements from 49 to 51 at% [28]. The investigated alloys were Ti_51-2x_Zr_x_Hf_x_Ni_49-2x_Cu_x_Co_x_ and Ti_49-2x_Zr_x_Hf_x_Ni_51-2x_Cu_x_Co_x_, with x = 1, 5, 10, and 17 at%. MT was observed only for x ≤ 5, while increasing the concentration of Ni-group elements decreased MTTs, consistent with the behavior of conventional TiNi-based alloys. In Ti_17_Zr_17_Hf_17_Ni_15_Cu_17_Co_17_, the B2 phase remained stable down to 173 K, and a perfect SE strain of approximately 2% was achieved.

The MTTs and functional properties of these alloys are summarized in Table 1 and Table 2, respectively.

**Table 1:** Martensitic transformation temperature (K) of TiZrHfNiCoCu alloys.

Alloy	Processing Heat Treatment	*M*_s_	*M*_f_	*A*_s_	*A*_f_	Thermal Hysteresis *A*_f_ − *M*_s_	Reference
Ti_16.67_Zr_16.67_Hf_16.67_Ni_25_Cu_25_	arc melting, as cast	500	385	458	611	111	[12]
Ti_16.67_Zr_16.67_Hf_16.67_Co_5_Ni_25_Cu_15_	arc melting, as cast	454	405	462	532	78	[13]
Ti_16.67_Zr_16.67_Hf_16.67_Co_10_Ni_25_Cu_15_	arc melting, as cast	430	373	423	488	58	[13]
Ti_16.67_Zr_16.67_Hf_16.67_Co_5_Ni_20_Cu_25_	arc melting, as cast	376	259	319	451	75	[13]
Ti_16.67_Zr_16.67_Hf_16.67_Co_10_Ni_20_Cu_10_	arc melting, as cast	263	200	240	317	54	[13]
Ti_16.67_Zr_16.67_Hf_16.67_Co_10_Ni_25_Cu_15_	Vacuum arc remelting (VAR), 1273 K 2 hWQ	309	193	250	344	35	[24]
Ti_16.67_Zr_16.67_Hf_16.67_Co_10_Ni_25_Cu_15_	VAR, 1173 K 24 h + 1223 K 12 h FC (FC)	276	225	253	310	34	[25]
Ti_16.67_Zr_16.67_Hf_16.67_Co_10_Ni_25_Cu_15_	VAR, 1273 K 2 h WQ	309	193	250	344	35	[25]
Ti_16.67_Zr_16.67_Hf_16.67_Co_10_Ni_25_Cu_15_	VAR, 1323 K 2 h (ST)	265	229	235	304	39	[26]
Ti_16.67_Zr_16.67_Hf_16.67_Co_10_Ni_25_Cu_15_	VAR, 1323 K 6 h(ST)	239	215	286	304	65	[26]
Ti_16.67_Zr_16.67_Hf_16.67_Co_10_Ni_25_Cu_15_	VAR, ST 2 h + 723 K 1.5 h WQ	225	205	269	235	10	[26]
Ti_48_Zr_1_Hf_1_Co_48_Ni_1_Cu_1_	arc melting, as cast	308	281	312	346	38	[27]

**Table 2:** Shape memory effect and superelasticity of TiZrHfNiCoCu.

Alloy	Processing Heat Treatment	SME Recovery Ratio	SE Recovery Ratio	Reference
Ti_16.67_Zr_16.67_Hf_16.67_Co_10_Ni_25_Cu_15_	VAR, 1273 K 2 h WQ	4.8%/650 MPa 94%	-	[24]
Ti_16.67_Zr_16.67_Hf_16.67_Co_10_Ni_25_Cu_15_	VAR, 1173 K 24 h + 1223 K 12 h FC	1.1%/200 MPa 95%	1.8%/RT 100%	[25]
Ti_16.67_Zr_16.67_Hf_16.67_Co_10_Ni_25_Cu_15_	VAR, 1273 K 2 h WQ	4.5%/650 MPa 92%	2%/RT 100%	[25]
Ti_16.67_Zr_16.67_Hf_16.67_Co_10_Ni_25_Cu_15_	VAR, 1323 K 2 h (ST)WQ	-	1.5%/RT 75% 2%/373 K 100%	[26]
Ti_16.67_Zr_16.67_Hf_16.67_Co_10_Ni_25_Cu_15_	VAR, ST 2 h + 723 K 1.5 hWQ	2.5% 100%	1%/RT 100% 2%/373 K 100%	[26]
Ti_17_Zr_17_Hf_17_Ni_15_Cu_17_Co_17_	arc melting, as cast		2%/173 K 100%	[28]

## 3. TiZrHfNiCu: B2-B19′

Because Co was identified as a strong B2 stabilizer that significantly suppresses MT and lowers MTT, subsequent research increasingly focused on Co-free TiZrHfNiCu alloys. The effects of Cu and Ni concentrations on MTT were systematically investigated in Ti_16.67_Zr_16.67_Hf_16.67_Ni_50-x_Cu_x_ alloys with x = 0, 5, 15, 25 [29]. As summarized in Table 3, increasing Cu content progressively decreased the MTT. In addition to transformation behavior, the thermal stability of these alloys was evaluated through repeated DSC measurements. Among the investigated compositions, Ti_16.67_Zr_16.67_Hf_16.67_Ni_35_Cu_15_ exhibited the highest thermal stability, showing the smallest change in MTT during thermal cycling. These results indicate that an appropriate balance between Ni and Cu is critical for achieving both desirable MTT and stable functional performance in TiZrHfNiCu HESMAs.

The effect of aging treatment on the microstructure and transformation behavior of the pseudo-equiatomic Ti_20_Zr_15_Hf_15_Ni_35_Cu_15_ alloy was systematically investigated [30]. This alloy composition satisfies the definition of HEA and exhibits extensive precipitation during aging. At aging temperatures below 773 K, the formation of the H phase was dominant. The composition of the H phase, characterized by a (Hf + Zr) content of 29.8 at%, and a (Ni): (Ti + Hf/Zr) ratio of 51.5:48.5, is similar to that reported in TiNiHf and TiNiZr alloys [31]. The elastic strain field around the H phase hindered MT, resulting in a reduction in MTT. With increasing aging temperature, the precipitation behavior changed significantly. Aging at 873 K produced a eutectoid structure consisting of Ti_2_Cu and (Zr, Hf)_7_Cu_10_ precipitates within the B19′ martensite matrix. The (Zr, Hf)_7_Cu_10_ phase was first identified in this alloy system. After aging at 973 K, both (Zr, Hf)_7_Cu_10_ and Ti_2_Ni phases were observed in the B19′ matrix, indicating further microstructural evolution at elevated temperatures.

The influence of aging on SE and elastocaloric effect (eCE) was subsequently investigated in Ti_30_Zr_10_Hf_10_Ni_35_Cu_15_, which exhibits an *A*_f_ below 273 K [32]. The solution-treated sample exhibited a higher *M*_s_, 263 K, than the sample furnace-cooled from 1223 K (FC), 219 K, while aging at 773 K reduced *M*_s_ because of the formation of coherent H-phase precipitates. In contrast, aging at 973 K increased *M*_s_ owing to the precipitation of coarse (Zr, Hf)_7_Cu_10_ particles, which depleted Cu from the matrix and consequently enriched the matrix in Ti. The resulting compositional change promoted MT and increased MTT. A notable feature of this alloy is the formation of H-phase even at a pseudo-binary composition with Ni + Cu = 50 at%, whereas H-phase precipitates in conventional TiNi-based alloys are generally associated with Ni-rich compositions. This observation suggests that Cu addition promotes H-phase formation and alters the precipitation behavior compared with conventional TiNi alloys. Ti_30_Zr_10_Hf_10_Ni_35_Cu_15_ exhibited excellent functional properties, including approximately 5% recoverable SE strain and an elastocaloric temperature change exceeding 14 K during reverse transformation. Furthermore, 75–77% of the initial eCE capacity was retained after 5000 loading-unloading cycles, demonstrating good functional stability. These results highlight the important role of precipitation in tailoring MTT and functional performance and suggest that TiZrHfNiCu are promising candidates for solid-state refrigerant applications.

The effect of nano precipitates on MT and SME was investigated in (Ti_40-x_Hf_10_Zr_x_)_50_(Ni_44_Cu_6_)_50_ derived from Ti_50_Ni_44_Cu_6_ [33]. In this study, Zr was added at concentrations of 1, 5, and 10 at%. All as-cast samples consisted primarily of the B2 phase, accompanied by Ti_2_Ni secondary phase. With increasing Zr content, additional Hf-rich or HfZr-rich phases were observed, indicating enhanced chemical partitioning in the MPEA. Following solution treatment at 1073 K, quenching in ice water, and subsequent aging at 573 or 673 K, nanoscale particles with sizes ranging from 50 to 80 nm were formed throughout the matrix. Based on compositional analysis, which yielded a (Ti + Zr + Hf):(Ni + Cu) ratio of 47.18:52.82, these precipitates were identified as H phase. Although the MTTs were not explicitly reported, the functional properties are summarized in Table 4. Compared with the as-cast TiNiCu ternary alloy, the as-cast HESMA exhibited enhanced SE, with further improvement after aging treatment. Among the investigated compositions, the alloy containing 5 at% Zr showed the most pronounced enhancement in SE. The improved SE behavior was attributed to the combined effects of severe lattice distortion arising from the MPEA and precipitation strengthening associated with the nanoscale H phase. These microstructural features increased both the yield strength and the critical stress required for stress-induced MT, thereby enabling larger recoverable strains. In particular, Zr addition markedly increased the yield strength and transformation stress, while the formation of nanoscale H-phase precipitates provided additional strengthening. These findings demonstrate that the synergistic effects of lattice distortion and nanoscale precipitation provide an effective strategy to improve the functional performance of HESMAs.

Several additional TiZrHfNiCu alloy compositions have also been investigated to clarify the effects of alloy composition and heat treatment on MT and functional properties [34,35,36,37]. One representative example is Ti_16_Zr_15_Hf_19_Ni_27_Cu_23_, which was homogenized at 1273 K for 190 h, solution treated at 1173 K for 2 h, and subsequently water quenched [34]. This alloy exhibited MTTs in the range of 400–523 K. Although MTTs were not explicitly reported, they were estimated from the DSC curves and are summarized in Table 3. Thermal cyclic tests demonstrated excellent stability of the transformation behavior, with only a slight decrease in MTT after 200 thermal cycles. In contrast, the ternary alloy Ni_49_Ti_33_Zr_19_ initially exhibited a larger recovery strain of 3.5%, but it degraded to 1.7% after repeated cycling. This comparison highlights the enhanced cyclic stability of the SME in HEAs.

The SE and SME of as-cast Ti_20_Zr_15_Hf_15_Ni_25_Cu_25_ were also evaluated [35]. The alloy exhibited approximately 4% perfect SE strain in the temperature range of 458–558 K and a recoverable strain for SME of 2.6% under an applied stress of 450 MPa, demonstrating excellent functional performance at elevated temperatures.

The influence of lower Zr and Hf contents was investigated in Ti_25_Zr_12.5_Hf_12.5_Ni_35_Cu_15_ [36]. This alloy exhibited high strength, favorable SME with a shape recovery ratio of 88.7% under an applied stress of 1400 MPa. The same alloys are homogenized at 1273 K for 4 h and compared with Ti_20_Zr_15_Hf_15_Ni_35_Cu_15_, with higher Zr and Hf contents [37]. Homogenization eliminated the dendritic microstructure and produced a more uniform microstructure, although the volume fraction of Ti_2_Ni precipitates increased. As summarized in Table 3, homogenization decreased MTT but also reduced thermal hysteresis. Comparison of these alloys suggests that increasing the Zr and Hf contents generally raises the MTT, whereas homogenization treatment tends to reduce MTT through changes in matrix composition associated with Ti_2_Ni precipitation. The compression test further revealed simultaneous improvements in strength and ductility after homogenization.

The influence of the Ni/Cu ratio on MTT, SE, and SME was investigated in Ti_25_Zr_10_Hf_15_Ni_28_Cu_22_ [38]. In this study, the Ni/Cu ratio was tailored. Compared with the equiatomic Ni/Cu alloy Ti_25_Zr_15_Hf_15_Ni_25_Cu_25_ [35], Ti_25_Zr_10_Hf_15_Ni_28_Cu_22_ exhibited a higher MTT and a relatively narrow thermal hysteresis of 41 K. In addition to its favorable transformation characteristics, the alloy demonstrated an excellent combination of strength and ductility, with an ultimate compressive strength of 1957 MPa and a fracture strain of 8%. The enhanced mechanical properties contributed to a remarkable SE of approximately 8% was achieved under an applied stress of 1200 MPa, which is significantly higher than the 4% SE strain reported for Ti_25_Zr_10_Hf_15_Ni_25_Cu_25_ [35].

The MTTs and functional properties of these alloys are summarized in Table 3 and Table 4, respectively.

**Table 3:** Martensitic transformation temperature (K) of TiZrHfNiCu alloys.

Alloy	Processing Heat Treatment	*M*_s_	*M*_f_	*A*_s_	*A*_f_	Thermal Hysteresis *A*_f_ − *M*_s_	Reference
Ti_16.67_Zr_16.67_Hf_16.67_Ni_50_	arc melting as cast 1st cycle	864	785	992	1068	204	[29]
Ti_16.67_Zr_16.67_Hf_16.67_Ni_45_Cu_5_	arc melting as cast 1st cycle	755	601	906	1016	261	[29]
Ti_16.67_Zr_16.67_Hf_16.67_Ni_35_Cu_15_	arc melting as cast 1st cycle	600	492	648	774	173	[29]
Ti_16.67_Zr_16.67_Hf_16.67_Ni_25_Cu_25_	arc melting as cast 1st cycle	470	438	516	562	92	[29]
Ti_20_Zr_15_Hf_15_Ni_35_Cu_15_	arc melting 1273 K 2 h WQ	454	392	436	495	41	[30]
Ti_30_Zr_10_Hf_10_Ni_35_Cu_15_	arc melting 1223 K 168 h FC (FC)	219	-	-	261	42	[32]
Ti_30_Zr_10_Hf_10_Ni_35_Cu_15_	arc melting 1273 K 2 h WQ (ST)	263	-	-	316	53	[32]
Ti_30_Zr_10_Hf_10_Ni_35_Cu_15_	arc melting ST + 773 K 2 h	233	-	-	283	50	[32]
Ti_30_Zr_10_Hf_10_Ni_35_Cu_15_	arc melting ST + 973 K 2 h	283	-	-	333	50	[32]
Ti_16_Zr_15_Hf_19_Ni_27_Cu_23_	CFSYS cold crucible in a levitation furnace 1273 K 190 h + 1173 K 2 h WQ	473	403	453	523	50	[34]
Ti_20_Zr_15_Hf_15_Ni_25_Cu_25_	arc melting, as cast	374	-	-	452	78	[35]
Ti_25_Zr_12.5_Hf_12.5_Ni_35_Cu_15_	arc melting, as cast	382	366	369	442	60	[36]
Ti_25_Zr_12.5_Hf_12.5_Ni_35_Cu_15_	arc melting, as cast	385	373	369	442	57	[37]
Ti_25_Zr_12.5_Hf_12.5_Ni_35_Cu_15_	arc melting, 1273 K 4 h	n/a	n/a	347	356		[37]
Ti_20_Zr_15_Hf_15_Ni_35_Cu_15_	arc melting, as cast	525	465	522	598	73	[37]
Ti_20_Zr_15_Hf_15_Ni_35_Cu_15_	arc melting, 1273 K 4 h	479	463	515	538	59	[37]
Ti_25_Zr_10_Hf_15_Ni_28_Cu_22_	arc melting, as cast	282	240	261	323	41	[38]

**Table 4:** Shape memory effect, superelasticity, and elastocaloric effect of TiZrHfNiCu alloys. IQ is iced quench, and AC is air cooling.

Alloy	Processing Heat Treatment	SME Recovery Ratio	SE Recovery Ratio	eCE (K)	Reference
Ti_30_Zr_10_Hf_10_Ni_35_Cu_15_	arc melting 1273 K 2 h WQ	-	4.6%/*A*_f_ + 50 K 92%	16	[32]
Ti_30_Zr_10_Hf_10_Ni_35_Cu_15_	arc melting 1273 K 2 h WQ aged 773 K	-	5%/*A*_f_ + 50 K 100%	14.8	[32]
Ti_30_Zr_10_Hf_10_Ni_35_Cu_15_	arc melting 1273 K 2 h WQ aged 973 K	-	5%/*A*_f_ + 50 K 100%	19.3	[32]
Ti_50_Ni_44_Cu_6_	arc melting, as cast		2.6%/n/a 52.3%		[33]
Ti_50_Ni_44_Cu_6_	arc melting 1073 K 0.5 h IQ + 573 K 2 h AC		3.9%/n/a		[33]
Ti_50_Ni_44_Cu_6_	arc melting 1073 K 0.5 h IQ + 673 K 2 h AC		4.2%/n/a		[33]
(Ti_40-x_Hf_10_Zr_x_)_50_(Ni_44_Cu_6_)_50_	arc melting, as cast		7.2–8.8%/n/a 75.5–77.8		[33]
(Ti_40-x_Hf_10_Zr_x_)_50_(Ni_44_Cu_6_)_50_	arc melting 1073 K 0.5 h IQ + 573 K 2 h AC		6.4–9.7%/n/a		[33]
(Ti_40-x_Hf_10_Zr_x_)_50_(Ni_44_Cu_6_)_50_	arc melting 1073 K 0.5 h IQ + 673 K 2 h AC		7.1–9.3%/n/a		[33]
Ti_16_Zr_15_Hf_19_Ni_27_Cu_23_	CFSYS cold crucible in a levitation furnace 1273 K 190 h + 1173 K 2 h WQ	2	-	-	[34]
Ti_20_Zr_15_Hf_15_Ni_25_Cu_25_	arc melting, as cast	2.6%/450 MPa 100%	2%/458–558 K		[35]
Ti_25_Zr_12.5_Hf_12.5_Ni_35_Cu_15_	arc melting, as cast	4.4/1400 MPa 88.7%	-	-	[36]
Ti_25_Zr_10_Hf_15_Ni_28_Cu_22_	arc melting, as cast	-	8%/RT 98.8%	7	[38]

## 4. Other TiZrHf or TiHf SMAs: B2-B19′

In addition to the widely investigated TiZrHfNiCu or TiZrHfNiCuCo HESMAs, several alternative compositional strategies have been explored to clarify the effects of elemental substitution on the MT behavior of TiZrHf-based HESMAs.

One representative approach involves the stepwise increase in alloy complexity from binary NiTi to multi-principal element alloys. As summarized in Table 5, a series of alloys, namely binary NiTi, ternary NiTiHf, quaternary NiCuTiHf, quinary NiCuTiZrHf, and senary NiCuPdTiZrHf, all exhibiting the B2 → B19′ MT, were systematically investigated [39]. The addition of Hf to binary NiTi markedly increased the MTT, with *M*_s_ increasing from 358.0 K to 697.2 K. However, further increases in configurational complexity did not result in a monotonic variation in MTT. Instead, *M*_s_ decreased to 317.4 K in the quaternary alloy, increased to 448.0 K in the quinary alloys, and decreased again to 332.6 K in the senary alloy, indicating that the MTT is not directly correlated with configurational entropy. The transformation behavior was further analyzed in terms of the valence electron concentration (C_V_). In general, a lower C_V_ corresponded to a higher *M*_s_ in NiTi-based alloys. Nevertheless, the quaternary and senary alloys deviated from this trend because Cu and Pd modify the relative stability of the B2 phase and the B19′/B19 martensite phases. In particular, Cu lowers the MTT, whereas Pd partially compensates for this effect. These results demonstrate that the influence of alloying elements on phase stability is more important than configurational entropy alone in determining the MTT.

The alloy-design strategy was further extended to off-stoichiometric NiCuPdTiZrHf alloys [40]. In this study, the ratio between the Ni equivalent (Ni, Cu, and Pd) and Ti equivalent (Ti, Zr, and Hf) elements was systematically varied while maintaining equal atomic fractions within each equivalent-element group. Although the off-stoichiometric compositions deviated from the conventional Cv-*M*_s_ relationship established for NiTi-based alloys, increasing the Ti-equivalent fraction shifted the alloys closer to this correlation. The highest MTT was obtained for Ni_13.333_Cu_13.333_Pd_13.333_Ti_20_Zr_20_Hf_20_, with an *M*_s_ of 570.7 K. Based on these results, it was proposed that maintaining equal atomic fractions within each equivalent-element group is essential for preserving the chemical stability of the B2 phase and promoting MT, even in highly multicomponent alloy systems. This concept provides a useful guideline for designing TiZrHf-based HESMA.

Another combinational strategy is represented by the (TiHfX)_50_(NiCu)_50_, where X = Y, Zr, Nb, or Cr [41]. Ti_35_Hf_10_X_5_Ni_44_Cu_6_ were prepared to investigate the effects of elemental substitution on the transformation behavior and SE. All alloys exhibited *M*_s_ below 273 K. The peak transformation temperatures (*M*_p_) of Y- and Zr-containing alloys were approximately 247 and 253 K, respectively. The martensite crystal structures were not identified in this study. Although the testing temperature was not explicitly reported, Y- and Zr-containing alloys exhibited excellent mechanical strength, reaching approximately 1500 MPa and 1700 MPa, respectively, at a pre-strain of 15%. In contrast, Nb- and Cr-containing alloys fractured before reaching 15% pre-strain, despite exhibiting strengths exceeding 1500 MPa. Cyclic loading tests conducted at a pre-strain of 6% further demonstrated stable SE for at least 20 loading-unloading cycles.

The MTTs and functional properties of these alloys are summarized in Table 5 and Table 6, respectively.

## 5. Quaternary-TiHf(Zr)Ni(Pt)Pd: B2-B19′/B19

The addition of Hf and Zr to TiNi has been extensively explored as an effective approach for increasing MTT, leading to the development of TiNiHf and TiNiZr high-temperature shape memory alloys (HTSMA) [42,43,44]. To expand ternary to quaternary alloys, Tobe et al. systematically investigated the phase transformation behavior of TiZrPdNi quaternary alloys [45]. Their work established comprehensive composition maps showing the martensitic crystal structures, H-phase formation regions, and MTT. The investigated compositions included Ti_30_Zr_20_(Ni_50-x_Pd_x_), with x = 5, 15, 25, 35, 45, together with several ternary and quaternary alloys such as Ti_25_Zr_25_Pd_50_, Ti_20_Zr_30_Pd_50_, Ti_15_Zr_35_Pd_50_, Ti_25_Zr_25_Ni_25_Pd_25_, Ti_15_Zr_35_Ni_25_Pd_25_. The results revealed a strong compositional dependence of martensitic structure and phase stability. In the Ti_30_Zr_20_(Ni_50-x_Pd_x_) series, Ti_30_Zr_20_Ni_49.7_ exhibited a B2 → B19′ MT, whereas Ti_35_Zr_15_Pd_49.7_ underwent a B2 → B19 MT. Intermediate compositions containing both Ni and Pd generally retained the B2 phase without a detectable MT except for Ti_30_Zr_20_Ni_44.7_Pd_5_ with B2-B19′ MT. In the ternary TiZrPd, Ti_10_Zr_40_Pd_49.7_ exhibited a B19′ or B33 martensitic structure, while Ti_35_Zr_15_Pd_49.7_ exhibited a B19 structure. These observations indicate that increasing Zr content stabilizes the B33 martensite, whereas increasing Ti content favors the B19 structure. In contrast, high Ni concentrations stabilize the B19′ structure. A notable feature of this alloy system is the existence of a broad compositional region in which the B2 phase remains stable. The suppression of MT in compositions located between the Ti-rich, Zr-rich, Pd-rich, and Ni-rich regions suggests that increasing compositional complexity and mixing entropy promote stabilization of the parent B2 phase. Following homogenization and cooling, short-range ordering was observed within the B2 matrix. Subsequent aging at 723–823 K for 3 h transformed this short-range ordered structure into the H-phase. H-phase was confirmed in T_30_Zr_20_Pd_50_, Ti_35_Zr_15_Pd_50_, and Ti_30_Zr_20_Ni_45_Pd_5_, demonstrating that aging-induced ordering is an important feature of this alloy. The MTT also showed a non-monotonic dependence on Pd concentration. Among the Ti_30_Zr_20_(Ni_50-x_Pd_x_) alloys, the highest *M*_s_ was observed in Ti_30_Zr_20_Ni_49.7_. The addition of 5 at% Pd reduced *M*_s_ from 573 K to 433 K in Ti_30_Zr_20_Ni_44.7_Pd_5_. For intermediate Pd concentrations of 15–45 at%, no MT was detected down to 133 K, indicating strong stabilization of the B2 phase. Interestingly, MT reappeared in the Pd-rich composition of Ti_30_Zr_20_Pd_49.7_, which exhibited an *M*_s_ of 413 K.

TiHfNiPd alloys have also been systematically investigated as a candidate for ultra-high-temperature SMA(HTSMAs) [46]. A particularly noteworthy finding was that the MTT increased significantly with increasing compositional complexity, progressing from ternary to quaternary and ultimately to quinary alloy. This trend contrasts with the MT suppression frequently observed in TiZrHfNiCu HESMA and highlights the unique role of alloy composition in governing phase stability and transformation behavior. It was found that the MTT increased drastically from ternary to quaternary to quinary with increasing entropy [46]. Although the MTTs were not explicitly summarized by the authors, they were estimated from the reported DSC curves and presented in Table 5. Among the investigated compositions, the equiatomic Ti_25_Hf_25_Ni_25_Pd_25_ exhibited an exceptionally high *M*_s_ of 893 K. Despite the remarkably high MTT, SME of Ti_25_Hf_25_Ni_25_Pd_25_ was limited to 0.8%, indicating that further optimization is required to improve functional performance. Nevertheless, Ti_25_Hf_25_Ni_25_Pd_25_ represents one of the earliest examples of an ultra-high-temperature high-entropy shape memory alloy and provides important insights into the design of SMA capable of operating at temperatures approaching 900 K.

TiZrPdPt has been systematically investigated in the TiPd-rich composition region, particularly for Zr contents up to 25 at% [47]. Following solution treatment at 1473 K for 3 h and subsequent water quenching, alloys containing 10 and 15 at% Zr exhibited a B2 → B19 MT, whereas no MT was observed in alloys containing 25 at% Zr. In general, the MTT decreased with increasing Zr content. The influence of Pt was less pronounced; although increasing Pt content raised the MT in the 5 at% Zr alloys, its effect became less significant at higher Zr concentrations. Microstructural observations revealed a typical martensitic twin structure in 10 and 15 at% Zr alloys. In contrast, 25 at% Zr alloys exhibited no martensitic twin structure. XRD analysis indicated Ti_25_Zr_25_Pd_25_Pt_25_ exhibited B2 + B33 structure, while the remaining 25 at% Zr alloys retained the B2 structure. These results indicate that increasing Zr content stabilizes the B2 phase, and the martensite structure changes to the B33 structure. Additional evidence for the stabilization of the B33 structure was obtained from furnace-cooled samples. In the 25 at% Zr alloys, slow cooling from 1473 K promoted the decomposition of the B2 phase and the formation of the B33 phase through a diffusion-assisted transformation process. An interesting feature of this alloy system is the pronounced supercooling effect observed in the 15 at% Zr alloys. MT occurred only when the alloys were cooled from sufficiently high temperatures, indicating that significant supercooling was required for the formation of martensitic twins. For example, Ti_35_Zr_15_Pd_35_Pt_15_ did not exhibit an exothermic transformation peak after cooling from 873 K, whereas a clear transformation peak appeared after cooling from 1073 K. The critical supercooling temperature increased systematically with Pt content, reaching 873 K, 1073 K, and 1273 K for Ti_35_Zr_15_Pd_45_Pt_5_ and Ti_35_Zr_15_Pd_35_Pt_15_, respectively. This trend suggests that increasing compositional complexity and mixing entropy suppress MT and increase the critical supercooling temperature.

Among the investigated compositions, only the 10 at% Zr alloys exhibited a measurable SME, whereas alloys containing 15 and 25 at% Zr showed little or no SME. Further studies demonstrated that reducing the Zr content to 5 at% increases the MTT and enables the formation of well-developed martensitic twin structure together with clear SME behavior [48,49]. These findings indicate that Zr plays a critical role in determining phase stability, transformation behavior, and functional properties in the TiZrPdPt system, with excessive Zr contents promoting B33 stabilization.

Although not classified as HEAs, several related quaternary alloys, including TiZrPdIr [8], TiZrVPd [50], TiZrPdNi [8,51], and TiZrPdCo [51], have also been investigated, and their MTTs are summarized in Ref. [8].

The MTTs and functional properties of these alloys are summarized in Table 7 and Table 8, respectively.

## 6. TiPd-Multi-Principal Element Alloys (MPEA):B2-B19

Although these alloys are generally classified as moderate-entropy alloys and multi-component alloys, several multi-principal-element alloys (MPEAs) based on the TiZrPd system have exhibited attractive functional properties. Representative examples include TiZrPdNiCo [51], TiZrPdPtNi, TiZrPdPtAu, and TiZrPdPtCo [52,53], all of which undergo a B2 → B19 MT. The effect of Ni and Co additions was systematically investigated in TiZrPdNiCo [51]. As summarized in Table 7, the addition of Ni and Co significantly altered the MTTs relative to the ternary alloy Ti_45_Zr_5_Pd_40_ (*M*_s_: 740, *M*_f_: 718, *A*_s_: 765, and *A*_f_: 781 K)/In particular, the equiatomic addition of 5 at% Ni and 5 at% Co results in a dramatic reduction in the MTT to below room temperature. In contrast, asymmetric additions of Ni and Co, such as Ti_45_Zr_5_Pd_40_Ni_8_Co_2_ and Ti_45_Zr_5_Pd_40_Ni_2_Co_8_, retained relatively high MTT. These results indicate that not only the total concentration of alloying elements but also their relative proportions play a critical role in determining phase stability and transformation behavior. The SME exhibited a similarly strong compositional dependence. As summarized in Table 8, Ti_45_Zr_5_Pd_40_Ni_8_Co_2_ and Ti_45_Zr_5_Pd_40_Ni_2_Co_8_ showed higher SME than quaternary Ti_45_Zr_5_Pd_40_Ni_10_. In contrast, Ti_45_Zr_5_Pd_40_Co_10_ exhibited poor SME because Co significantly reduced the transformation strain. Notably, Ti_45_Zr_5_Pd_40_Ni_2_Co_8_ maintained a relatively large SME despite containing only 2 at% Ni, suggesting that even a small amount of Ni can effectively enhance the transformation strain and functional response.

The combination of Pd and Pt generally produces high MTTs in TiZrPdPt-based alloys. However, the addition of 15 at% Au, Co, or Ni, corresponding to a mixing entropy of ∆S = 1.5R, completely suppressed MT, and not MT was detected within the investigated temperature range [38,39]. In contrast, when the total addition of the fifth element was below 10 at% (∆S < 1.3R), clear MT was observed, with *M*_s_ ranging from 610 to 787 K. Ti_45_Zr_5_Pd_20_Pt_25_Ni_5_ and Ti_45_Zr_5_Pd_20_Pt_20_Ni_10_ exhibited SME of 1.8 and 2.3%, respectively [52]. Repeated thermal cyclic tests, called training, effectively reduced the irrecoverable strain and enabled nearly perfect shape recovery. For Ti_45_Zr_5_Pd_20_Pt_25_Ni_5_, complete recovery strains of 1.6% and 1.0% were achieved under applied stresses of 200 and 300 MPa, respectively. Similarly, Ti_45_Zr_5_Pd_20_Pt_20_Ni_10_ exhibited perfect recovery of 1.5% at 200 MPa and 0.5% at 300 MPa. Although complete recovery was maintained, the recoverable strain decreased with increasing applied stress. The effects of Au and Co additions were also investigated [53]. Alloys containing 5 at% Au and Co exhibited recoverable strain of 2 and 1.2%, respectively, under an applied stress of 200 MPa. Notably, the Co-containing alloy achieved complete recovery after 83 training cycles, whereas the Au-containing alloy retained a measurable irrecoverable strain even after training. While Co addition improved cyclic stability, it also significantly reduced the transformation strain.

The effect of V on MT, SME, SE, and eCE was investigated in Ti_50-x_V_x_Pd_25_Ni_15_Cu_10_ with x = 0, 0.5, 1.0, 1.5 [54]. All alloys underwent a B2 → B19 MT, and fine TiPdCu precipitates were formed during aging. Since only the *M*_s_ was explicitly reported, the MTT summarized in Table 7 is limited to *M*_s_. Both V addition and aging treatment reduced *M*_s_, which was attributed to the strain fields associated with coherent TiPdCu precipitates that hindered MT. The results further suggested that V promotes the formation of the TiPdCu phase, thereby enhancing the suppression of MT. In contrast, aging at a higher temperature of 973 K produced a much smaller reduction in *M*_s_. This behavior was attributed to precipitate coarsening and the loss of coherence between the precipitates and the matrix, which reduced the associated strain fields and their influence on MT. The SME were strongly dependent on the aging condition. The largest recoverable strain was obtained in 1.5 V alloy aged at 573 K for 1 h, while the irrecoverable strain remained remarkably low ats 0.1~0.2%, indicating nearly perfect shape recovery. In contrast, the lowest recovery strain was obtained in 1.5 V alloy aged at 973 K for 4 h, suggesting that coarse precipitates are less effective in promoting MT than fine coherent precipitates. SE also benefited from precipitation control. Complete strain recovery of 2% was obtained under solution-treated conditions, whereas the alloy aged at 573 K for 1 h exhibited a significantly improved SE of 4%. The elastocaloric effect (eCE) was evaluated under pre-strains of 4% and 5%, yielding temperature changes of 11.3 and 12.5 K, respectively. These results demonstrate that coherent nanoscale precipitates can effectively enhance SME, SE, and eCE.

TiV(Zr)PdPtNi alloys were designed as intermediate compositions, similar to the TiPdNiCu [54] and TiZrHfPdPtCo [53], with the objective of simultaneously controlling MTT and transformation strain [55]. Systematic investigations revealed that alloy composition profoundly influences phase stability and transformation behavior. For example, increasing the V content from 1.5 to 5 at% significantly suppressed MT, as demonstrated by the comparison between Ti_48.5_V_1.5_Pd_25_Pt_10_Ni_15_ and Ti_45_V_5_Pd_25_Pt_10_Ni_15_. However, in alloys containing 5 at% V, partial substitution of Pd and Ni by Pt restored the MT, resulting in MTTs above room temperature and *M*_s_ as high as 773 K. These observations highlight the strong sensitivity of transformation behavior to the balance among constituent elements. The individual alloying elements were found to play distinct roles in determining functional properties. Ni generally improved SME, although excessive Ni addition lowered the MTT. Zr enhances high-temperature strength but tends to suppress MT when added in large amounts. This behavior is associated with the competing transformation pathways present in the TiZrPd alloys. While TiPd-rich alloys undergo a B2 → B19 MT, PdZr-rich compositions exhibit a B2 → B33 transformation. Increasing the Zr content therefore promotes B33 stabilization, leading to competition between the B19 and B33 martensitic structures and suppression of the B2 → B19 transformation. An additional indication of entropy effects was observed in Ti_45_Zr_5_Pd_16.6_Pt_16.8_Ni_16.6_, in which Pd, Ni, and Pt were present in equiatomic proportion. Despite its compositional similarity to Ti_45_Zr_5_Pd_20_Pt_20_Ni_10_, this alloy exhibited a lower MTT. The result suggests that increasing mixing entropy can stabilize the parent phase and reduce MTT. This trend contrasts with the behavior reported by Canadinc’s [46], where equiatomic TiZrHfNiPd resulted in higher MTT, emphasizing that the influence of entropy on MT is strongly dependent on alloy composition. Most of the investigated alloys exhibited recoverable strains of approximately 2% under an applied stress of 200 MPa and showed nearly complete SME over a wide MTT range of 393–633 K. An exception was Ti_45_V_5_Pd_20_Pt_20_Ni_10_, which exhibited MTT between 673 and 898 K, and a large irrecoverable strain was observed. Although such high MTTs are attractive for HTSMAs, the alloy shows reduced functional performance because plastic deformation becomes more pronounced at elevated temperatures.

The effect of operating temperature on the functional stability of Ti_45_Zr_5_Pd_25_Pt_19_Co_6_ exhibiting a B2 → B19 MT was systematically investigated [56]. Cyclic DSC measurements were performed over two temperature ranges, 473–673 K and 473–773 K, to evaluate the stability of transformation behavior during repeated thermal cycling. Remarkably, the alloy cycled between 473 and 773 K exhibited significantly greater thermal stability than the alloy cycled between 473 and 673 K. After five thermal cycles, the decrease in the peak transformation temperature, *M*_p_ (*M*_s_ + *M*_f_/2), was 0.5 K for the 473–773 K cycle, whereas a reduction of 12.4 K was observed for the 473–673 K cycle. Similarly, *M*_s_ remained essentially unchanged during cycling to 773 K but decreased by 20.9 K during cycling to 673 K. The SME and SE exhibited similar trends. The alloy cycled to 773 K maintained stable SME and SE during repeated cycling, whereas a greater degradation of SME and SE was observed for the alloy cycled to 673 K. The enhanced functional stability can be understood in terms of the crystallographic compatibility and recovery of deformation defects. The alloy exhibits a middle eigenvalue (λ2) of 1.04, indicating imperfect compatibility between the martensite and austenite phases. Consequently, interfacial stresses generated during MT produce dislocations that accumulate during thermal cycling. When the operating temperature is limited to 673 K, these dislocations remain largely unrecovered, resulting in progressive degradation of transformation behavior. In contrast, cycling to 773 K provides sufficient thermal activation for dislocation recovery and annihilation, thereby reducing defect accumulation and stabilizing the transformation characteristics. A particularly important finding is that this self-recovery mechanism operates in a high-strength multi-principal element-SMA.

The MTTs and functional properties of these alloys are summarized in Table 9 and Table 10, respectively.

## 7. Comparison of HESMAs

**Figure 1:**
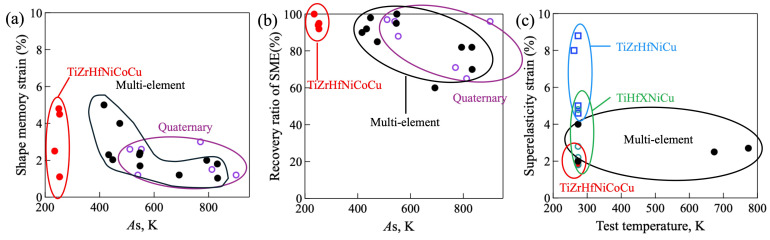
(**a**) Shape memory strain, (**b**) recovery ratio of shape memory effect, and (**c**) superelasticity strain of HESMAs. Red: TiZrHfNiCoCu (Section 2), Blue: TiZrHfNiCu (Section 3), Green: TiHfXNiCu (Section 4), Purple: Quaternary alloys (Section 5), Black: Multi-element alloys (Section 6).

The shape memory strain, shape recovery ratio, and superelasticity (SE) strain reported for TiZrHf-based HESMAs are summarized in Figure 1. The shape memory strain and recovery ratio of SME are plotted as a function of *A*_s_, whereas the SE strain is plotted against the test temperature.

A wide range of MTTs has been achieved in the quaternary and multi-principal element alloys reviewed in this work. In particular, several alloys exhibit *A*_s_ above 800 K, whereas *A*_s_ of TiZrHfNiCoCu is generally around 473 K. The shape memory strain strongly depends on alloy composition, with a maximum recoverable strain of approximately 5%. In the multi-principal element and quaternary alloys, the shape memory strain generally decreases with increasing *A*_s_. Likewise, the shape recovery ratio remains close to 100% for alloys with *A*_s_ below approximately 400 K but gradually decreases as *A*_s_ increases, indicating that plastic deformation becomes more significant at elevated MTT.

Because the alloys investigated for SE generally have MTT below room temperature, the SE strain is compared as a function of the testing temperature. Most alloys were evaluated at room temperature and exhibited nearly complete strain recovery. TiZrHfNiCu showed relatively large SE strains of approximately 4–9%, whereas TiZrHfNiCoCu exhibited SE strains of only about 2%. This comparison suggests that Co addition significantly decreases both the MTT and the recoverable SE strain. In contrast, multi-principal element alloys generally exhibit moderate SE strains of 2–4%; however, nearly complete recovery can be maintained even at elevated testing temperatures. These results suggest that increasing compositional complexity is beneficial for improving the high-temperature SE response, although the maximum recoverable strain is somewhat reduced.

The effect of the multi-principal alloying on MTT is considerably more complex than that of conventional alloying. In ternary NiTi alloys, *M*_s_ generally follows the well-established correlation with the valence electron concentration (Cv), with lower Cv corresponding to higher *M*_s._ However, this relationship no longer holds for quaternary and higher-order alloys, such as NiTiCuPdZrHf, which deviate significantly from the conventional Cv-*M*_s_ correlation. The deviation reflects the competing roles of different alloying elements: Cu destabilizes the martensite phase and lowers *M*_s_, whereas Pd has the opposite effect and increases *M*_s_. Consequently, the transformation behavior of multi-principal element alloys cannot be interpreted using a single empirical parameter. Instead, reliable alloy design requires a comprehensive understanding of the individual effects of each alloying element on phase stability, as well as their collective interactions in multicomponent alloys.

## 8. Machine Learning (ML)

The design of HE-SMAs and MPESMA is considerably more complex than that of conventional binary or ternary SMAs due to the larger compositional space. In recent years, machine learning (ML) has emerged as a promising tool for accelerating alloy design and optimizing functional properties.

Xue et al. developed an adaptive alloy-design framework to identify alloy compositions exhibiting low thermal hysteresis in Ti_50_Ni_50-x-y-z_Cu_x_Fe_y_Pd_z_ alloys [57]. Using a dataset consisting of 22 experimentally characterized alloys, several physically meaningful descriptors, including Valence electron number, Pauling electronegativity, pseudopotential radius, metallic radius, atomic radius, and Pettifor chemical scale, were employed as input features for machine learning models. These descriptors were selected based on their known relationships with phase transformation behavior; for example, MTT is strongly correlated with valence electron concentration (VEC), whereas thermal hysteresis is influenced by atomic size mismatch and lattice distortion [58]. Several regression algorithms, including Gaussian Process Model (GPM), SVRrbf, and SVRlin, were employed to explore a compositional space containing approximately 7.97 × 10^5^ candidate compositions generated by systematically varying the Cu, Fe, and Pd contents. Bayesian-based approaches enabled efficient exploration of the design space by balancing exploitation of promising compositions with exploration of unexplored regions. The framework identified Ti_50_Ni_46.7_Cu_0.8_Fe_2.3_Pd_0.2_ as the optimal composition with a predicted thermal hysteresis of only 1.84 K. To further elucidate the underlying mechanism, density functional theory (DFT) calculations were combined with machine learning. The results revealed that thermal hysteresis decreases as the energy difference between austenite and martensite approaches 0, indicating that a smaller thermodynamic barrier facilitates reverse MT.

Machaka et al. developed an interpretable machine learning framework to predict MTT of TiNi-based HTSMA [59]. The study utilized a dataset comprising 396 experimental measurements collected from 113 Ti-Ni-based alloys reported in the literature. Due to the limited datasets, dimensionality reduction was performed using sequential backward and forward feature selection algorithms, and only alloy composition-based descriptors were retained for model development. A wide range of machine learning algorithms was evaluated, including the multi-linear regression (MLR), the ridge regression (RRR), the support vector machine regression (SVR), K-nearest neighbors (KNN), the Least Absolute Shrinkage and Selection Operator (LASSO), the simple decision tree (DT), and its modifications including the decision tree bagging or the Random Forest (RF), the family of boosted decision trees including the adaptive boosting (AdaBoost), and the extreme gradient boosting (XGBoost), the light gradient boosting machine (LGBM) and the CatBoost classifier and regressor (CT), and the multilayer perceptron artificial neural network regressor (ANN). The predictive capabilities of these models were further validated using newly prepared alloys that were not included in the training dataset. The decision tree-based regression algorithms RF, XGBoost, and CT posted significantly better performance in thermal hysteresis. XGBoost-based model provided the highest accuracy, with prediction errors below 3%, and was particularly effective in predicting thermal hysteresis. The model was interpreted using SHapley Additive exPlanations (SHAP) analysis. It was also demonstrated that substituting Ti with Cu significantly narrows thermal hysteresis. Substituting Ni with Zr significantly widens the thermal hysteresis. This study serves as a representative example of the full-scale implementation of SHAP in HESMA research. Machine learning offers a deeper understanding of how the input materials’ composition descriptors can contribute to hysteresis.

He et al. developed an interpretable machine learning framework to predict the martensitic transformation peak temperature (*T*_p_) of TiZrHfNiCoCu HE-SMAs [60]. In total, 50 sets of experimental data were used from the same batch. An interpretable machine learning workflow was established through dataset construction, feature selection, modeling, validation, and model interpretation. Feature selection was performed using Pearson correlation analysis, univariate feature selection, and forward feature elimination. The resulting feature set was used to construct predictive models, which were validated using three newly synthesized alloys. The ML model using SVR performed better. The prediction errors were below 3%, demonstrating excellent predictive capability. A key innovation was the application of SHAP to interpret model behavior. SHAP analysis revealed that the Allred–Rochow electronegativity descriptor was the most influential variable governing transformation temperature. The average lattice parameter and deviation of lattice parameter also affect *T*_p_. TiZrHfNiCoCu containing 9 ≤ Co ≤ 10 and 15 ≤ Cu ≤ 17 at% have a pronounced positive effect on *T*_p_.

Tian et al. established the workflow to predict Martensitic transformation peak temperature (*M*_p_) and thermal hysteresis (*T*_hy_) [61]. They focused on TiZrHf-HEA. They created a dataset containing 33,264 data points using an A_50_B_50_ structure. The composition is the content of A elements, such as Ti, Zr, and Hf, to 10 < X < 20%, and the content of class B elements, such as Ni, Co, Cu, Pd, Pt, and Fe, to 5 < X < 35%. SHAP analysis indicated that the enthalpy of missing (H), Deviation of Young Modulus (Cym), Deviation of lattice parameter a (Ca), lattice constant a, Deviation of Pauling electronegativity (Cen), and Valence electron numbers (Vec) are the most important features for *M*_p_. For *T*_hy_, Cen, Cym, Lattice constant c, Lattice constant a, and Deviation of lattice constant c (Cc) are the most important features. Feature engineering combined correlation screening and BorutaShap feature selection. Multiple machine learning algorithms were evaluated, such as Random Forest, decision tree, LightGBM, Linear Regression, and Extreme Gradient Boosting Regression (XGBR). XGBR exhibited the best performance. The *M*_p_ and *T*_hy_ models are trained by the XGBR combined with an optimization algorithm. The XGBR model demonstrates superior performance, achieving R^2^ test values of 0.902 for *M*_p_ and 0.928 for *T*_hy_. However, there is limited data containing Pt, Fe, Mn, and Cr in this dataset; these features contribute little to the model. SHAP analysis indicates that Hf, Pd, and Zr additions increase *M*_p_, while Cu and Co additions decrease *M*_p_. The effect of Cen, Ca, H, Vec, Cym, and the start equation a is more complicated. When Cen is low, the influence on the *M*_p_ is negative; that is a decrease in *M*_p_. Ca and a mainly reflect the degree of lattice distortion. SHAP value increases with the increase in Ca and decreases with the increase in a. Strongly negative H indicates formation of a stable solid solution, and a positive H indicates unmixing. The lower the H value, the higher the SHAP value. SHAP analysis indicates which feature affects *M*_p_. SHAP analysis was also performed for *T*_hy_. A decrease in Ti content increases the SHAP value, indicating a wider *T*_hy_. This is due to the lattice distortion caused by the increase in Zr and Hf by decreasing Ti. High Cu content has a negative effect on *T*_hy_ because Cu addition improves the interfacial compatibility between austenite and martensite, enhancing the functional stability.

Thiercelin et al. developed a physics-informed machine learning (PIML) framework for predicting the *M*_s_ of NiTi-like HE-SMA [62]. The data set was significantly expanded to include binary, ternary, quaternary, quinary, and senary alloys containing Ni-equivalent and Ti-equivalent elements. The database consists of 396 alloys, including 240 binary and ternary alloys, 113 quaternary alloys, and 43 HEAs. The machine learning model employed the Extremely Randomized Trees (Etra Trees) algorithm. Two input strategies were compared. The first one is alloy compositions as predictors, whereas the second is physically meaningful descriptors such as atomic radius, electronegativity, atomic number, misfit energy, and number of constituent elements. Physical material descriptors are useful for the prediction of *M*_s_. At least six parameters are sufficient for having a good *M*_s_ prediction. It also suggested that physical material descriptors provided some physical properties to better understand the effect of specific elements. For instance, replacing Ni with Pd increases *M*_s_. It is suggested that this is due to the proximity of Pd and Ni, combined with a higher atomic number, higher electronegativity, and higher mixing enthalpy. It was also reported that as the number of elements increases, the error increases, especially when it exceeds 5. The assumption is that homogeneity is difficult for HEA, causing *M*_s_ to depend on the preparation process.

Sridharan et al. focused on high-temperature SMAs consisting of Ti-Zr-Hf-Pd-Pt to predict martensitic temperature (*M*_m_), austenitic temperature (*A*_m_), and thermal hysteresis (*T*th) [63]. Three ensemble-learning models were developed. Model M1 used only elemental compositions as input. Model M2 used material descriptors derived from thermodynamic, structural, and electronic properties. Model M3 combined alloy compositions and material descriptors. Bayesian optimization and cross-validation were used to optimize model parameters and ensemble weights. Model M1 predicted *M*_m_ and *A*_m_ with higher accuracy. Models M2 and M3 were slightly less accurate, but they are useful for providing insights into the mechanism underlying the phase transformation behavior. Enthalpy of mixing, electronegativity, valence electron concentration, density-based parameter, deviation in atomic radius, and ideal entropy of mixing significantly influenced MTT and *T*th. Feature importance analysis indicates the relevance of the alloying element. Pt and Pd are effective in raising MTT and *T*th because of the bonding characteristics and stability of the austenitic phase. Hf and Zr are comparatively insignificant, but they have an effect of increasing MTT and decreasing *T*th.

The research on ML to predict MTT has increased to design HESMA. Not only the prediction of MTT, but the understanding of the phase transformation mechanism also plays an important role in ML. It is evident that research on HESMA is currently shifting from high-precision prediction to explainable alloy design. At the moment, the prediction model for SME and SE is limited. The experimental data of SME and SE are varied in different literature. Then, it is more difficult to collect data.

## 9. Conclusions

This review summarizes recent advances in Ti-based high-entropy and multi-principal-element shape memory alloys exhibiting B2 → B19′ and B2 → B19 martensitic transformations. Zr and Hf are primarily substituted for Ti, whereas Cu, Co, and Pd are substituted for Ni. Among the reported high-entropy shape memory alloys, (TiZrHf)(NiCuCo) alloys have been the most extensively investigated. The effects of the Ni/Cu ratio and nano precipitates on transformation behavior and functional properties have also been studied. Their excellent elastocaloric performance highlights their potential for solid-state cooling applications. TiHf(Zr)Pd(Pt)Ni has emerged as a promising high-temperature shape memory alloy, while TiPd-based multi-principal-element alloys also show considerable potential for high-temperature applications. Martensitic transformation temperature and thermal hysteresis are key parameters in SMA design, and machine learning approaches have recently been employed to predict these properties. Furthermore, the combination of machine learning and SHAP analysis enables not only the prediction of MTT and thermal hysteresis but also a deeper understanding of the factors governing phase transformation behavior.

## Figures and Tables

**Table 5:** Martensitic transformation temperature (K) of TiZrHf or TiHf alloys.

Alloy	Processing Heat Treatment	*M*_s_	*M*_f_	*A*_s_	*A*_f_	Thermal Hysteresis *A*_f_ − *M*_s_	Reference
Ni_50_ Ti_50_	arc melting 1173 K 100 h WQ	358	322.2	356.6	389.3	31.3	[39]
Ni_50_ Ti_25_Hf_25_	arc melting 1173 K 100 h WQ	697.2	643.8	688.6	714.4	17.2	[39]
Ni_25_Cu_25_Ti_25_Hf_25_	arc melting 1173 K 100 h WQ	317.4	297.6	332.3	354.8	37.4	[39]
Ni_25_Cu_25_Ti_16.6_Zr_16.6_Hf_16.6_	arc melting 1173 K 100 h WQ	448.0	410.2	458.4	494.2	46.2	[39]
Ni_16.6_Cu_16.6_Pd_16.6_Ti_16.6_Zr_16.6_Hf_16.6_	arc melting 1173 K 100 h WQ	332.6	303.8	334.8	375.1	42.5	[39]
Ni_17_Cu_17_Pd_17_Ti_16.333_Zr_16.333_Hf_16.333_ NiCuPd:TiZrHf = 51:49 SenΔ1	arc melting 1173 K 100 h WQ	229.8	196.4	249.0	290.1	60.3	[40]
Ni_15.833_Cu_15.833_Pd_15.833_Ti_17.5_Zr_17.5_Hf_17.5_ NiCuPd:TiZrHf = 47.5:52.5 SenΔ-2.5	arc melting 1173 K 100 h WQ	382.7	346.0	401.1	429.0	46.3	[40]
Ni_15_Cu_15_Pd_15_Ti_18.333_Zr_18.333_Hf_18.333_ NiCuPd:TiZrHf = 45:55 SenΔ-5	arc melting 1173 K 100 h WQ	482.4	445.5	508.5	550.9	68.5	[40]
Ni_13.333_Cu_13.333_Pd_13.333_Ti_20_Zr_20_Hf_20_ NiCuPd:TiZrHf = 40:60 SenΔ-10	arc melting 1173 K 100 h WQ	570.7	535.8	804.0	829.9	259.2	[40]

**Table 6:** Superelasticity of TiZrHf or TiHf alloys.

Alloy	Processing Heat Treatment	SE Recovery Ratio	Reference
Ti_35_Hf_10_Zr_5_Ni_44_Cu_6_	arc melting as cast	4.8%/n/a 95%	[41]
Ti_35_Hf_10_Y_5_Ni_44_Cu_6_	arc melting as cast	2.8%/n/a 92%	[41]
Ti_35_Hf_10_Nb_5_Ni_44_Cu_6_	arc melting as cast	1.8%/n/a 95%	[41]
Ti_35_Hf_10_Cr_5_Ni_44_Cu_6_	arc melting as cast	2.2%/n/a 95%	[41]

**Table 7:** Martensitic transformation temperature (K) of TiHf(Zr)Ni(Pt)Pd.

Alloy	Processing Heat Treatment	*M*_s_	*M*_f_	*A*_s_	*A*_f_	Thermal Hysteresis *A*_f_ − *M*_s_	Reference
Ti_30_Zr_20_Ni_50_	arc melting 1323 K 24 h WQ	573	-	593	-	-	[45]
Ti_30_Zr_20_Ni_45_Pd_5_	arc melting 1323 K 24 h WQ	523	-	453	-	-	[45]
Ti_30_Zr_20_Ni_35_Pd_15_	arc melting 1323 K 24 h WQ	<133	<133	<133	<133		[45]
Ti_30_Zr_20_Ni_15_Pd_35_	arc melting 1323 K 24 h WQ	<133	<133	<133	<133	-	[45]
Ti_30_Zr_20_Ni_5_Pd_45_	arc melting 1323 K 24 h WQ	<133	<133	<133	<133	-	[45]
Ti_30_Zr_20_Pd_50_	arc melting 1323 K 24 h WQ	413	-	423	-	-	[45]
Ti_30_Hf_20_Pd_15_Ni_35_	arc melting 1323 K 2 h WQ	798	752	810	959	161	[46]
Ti_25_Hf_25_Pd_25_Ni_25_	arc melting 1323 K 2 h WQ	893	853	953	993	100	[46]
Ti_16.6_Zr_16.6_Hf_16.7_Pd_25_Ni_25_	arc melting 1323 K 2 h WQ	923	893	1003	1053	130	[46]
Ti_35_Zr_15_Pd_45_Pt_5_	arc melting 1473 K 3 h WQ	-	-	514	555	-	[47]
Ti_35_Zr_15_Pd_35_Pt_15_	arc melting 1473 K 3 h WQ	-	-	503	547	-	[47]
Ti_35_Zr_15_Pd_25_Pt_25_	arc melting 1473 K 3 h WQ	-	-	519	567	-	[47]
Ti_40_Zr_10_Pd_45_Pt_5_	arc melting 1473 K 3 h WQ	554	525	553	583	29	[47]
Ti_40_Zr_10_Pd_35_Pt_15_	arc melting 1473 K 3 h WQ	507	466	511	538	31	[47]
Ti_40_Zr_10_Pd_25_Pt_25_	arc melting 1473 K 3 h WQ	517	473	540	576	59	[47]
Ti_45_Zr_5_Pd_45_Pt_5_	arc melting 1273 K 3 h WQ	737	728	770	784	47	[48]
Ti_45_Zr_5_Pd_35_Pt_15_	arc melting 1273 K 3 h WQ	758	744	812	830	72	[48]
Ti_45_Zr_5_Pd_25_Pt_25_	arc melting 1273 K 3 h WQ	844	812	901	921	77	[48]
Ti_45_Zr_5_Pd_15_Pt_35_	arc melting 1273 K 3 h WQ	986	863	998	1077	91	[49]
Ti_45_Zr_5_Pd_5_Pt_45_	arc melting 1273 K 3 h WQ	1047	1208	1169	986	112	[49]
Ti_45_Zr_5_Pd_45_Ni_5_	arc melting 1273 K 3 h WQ	601	570	621	651	50	[49]

**Table 8:** Shape memory effect of quaternary TiHf(Zr)Ni(Pt)Pd.

Alloy	Heat Treatment	SME	Recovery Ratio	Reference
Ti_30_Hf_20_Pd_15_Ni_35_	arc melting 1323 K 2 h WQ	0.8%/70 MPa		[46]
Ti_40_Zr_10_Pd_45_Pt_5_	arc melting 1473 K 3 h WQ	2.6%/200 MPa	88%	[47]
Ti_40_Zr_10_Pd_35_Pt_15_	arc melting 1473 K 3 h WQ	2.6%/200 MPa	97%	[47]
Ti_40_Zr_10_Pd_25_Pt_25_	arc melting 1473 K 3 h WQ	1.2%/200 MPa	96%	[47]
Ti_45_Zr_5_Pd_45_Pt_5_	arc melting 1273 K 3 h WQ	3%/200 MPa	71%	[48]
Ti_45_Zr_5_Pd_25_Pt_25_	arc melting 1273 K 3 h WQ	1.5%/200 MPa	65%	[48]
Ti_45_Zr_5_Pd_45_Ni_5_	arc melting 1273 K3 h WQ	10%/200 MPa	99.8%	[49]

**Table 9:** Martensitic transformation temperature (K) of TiPd-multi-principal element alloys.

Alloy	Processing Heat Treatment	*M*_s_	*M*_f_	*A*_s_	*A*_f_	Thermal Hysteresis *A*_f_ − *M*_s_	Reference
Ti_45_Zr_5_Pd_40_Ni_8_Co_2_	arc melting 1273 K 3 h WQ	485	472	474	546	61	[51]
Ti_45_Zr_5_Pd_40_Ni_5_Co_5_	arc melting 1273 K 3 h WQ	-	-	-	-	-	[51]
Ti_45_Zr_5_Pd_40_Ni_2_Co_8_	arc melting 1273 K 3 h WQ	408	381	416	462	54	[51]
Ti_40_Zr_10_Pd_20_Pt_15_Ni_15_	arc melting 1273 K 3 h WQ	-	-	-	-	-	[52]
Ti_45_Zr_5_Pd_20_Pt_25_Ni_5_	arc melting 1273 K 3 h WQ	775	705	832	871	96	[52]
Ti_405_Zr_5_Pd_20_Pt_20_Ni_10_	arc melting 1273 K 3 h WQ	610	529	546	715	185	[52]
Ti_35_Zr_15_Pd_20_Pt_15_Au_15_	arc melting 1273 K 3 h WQ	-	-	-	-	-	[52]
Ti_35_Zr_15_Pd_20_Pt_15_Co_15_	arc melting 1273 K 3 h WQ	-	-	-	-	-	[53]
Ti_45_Zr_5_Pd_25_Pt_20_Au_5_	arc melting 1273 K 3 h WQ	787	726	793	863	75	[53]
Ti_45_Zr_5_Pd_25_Pt_20_Co_5_	arc melting 1273 K 3 h WQ	704	597	692	811	107	[53]
Ti_50_Pd_25_Ni_15_Cu_10_	arc melting 1223 K 168 h FC + 1273 K 2 h WQ (ST)	447					[54]
Ti_50_Pd_25_Ni_15_Cu_10_	arc melting ST + 573 1 h WQ	445					[54]
Ti_48.5_V_1.5_Pd_25_Ni_15_Cu_10_	arc melting 1223 K 168 h FC + 1273 K 2 h WQ (ST)	336					[54]
Ti_48.5_V_1.5_Pd_25_Ni_15_Cu_10_	arc melting ST + 573 K 1 h WQ	276					[54]
Ti_48.5_V_1.5_Pd_25_Ni_15_Cu_10_	arc melting ST + 573 K 672 h WQ	-	-	-	-		[54]
Ti_48.5_V_1.5_Pd_25_Ni_15_Cu_10_	arc melting ST + 973 K 8 h WQ	331					[54]
Ti_48.5_V_1.5_Pd_25_Pt_10_Ni_15_	arc melting 1473 K 2 h WQ	438	393	433	483	45	[55]
Ti_45_V_5_Pd_25_Pt_10_Ni_15_	arc melting 1473 K 2 h WQ	-	-	-	-		[55]
Ti_45_V_5_Pd_20_Pt_20_Ni_10_	arc melting 1473 K 2 h WQ	773	673	833	898	125	[55]
Ti_45_Zr_5_Pd_20_Pt_20_Ni_10_	arc melting 1473 K 2 h WQ	593	563	588	633	40	[55]
Ti_45_Zr_5_Pd_16.6_Pt_16.8_Ni_16.6_	arc melting 1473 K 2 h WQ	443	423	448	493	50	[55]
Ti_45_Zr_5_Pd_25_Pt_19_Co_6_	arc melting 1473 K 3 h WQ	617	520	548	648	31	[56]

**Table 10:** Shape memory effect and erastcaloric effect of TiPd-multi-principal element alloys.

Alloy	Processing Heat Treatment	SME Recovery Ratio	SE	eCE (K)	Reference
Ti_45_Zr_5_Pd_40_Ni_8_Co_2_	arc melting 1273 K 3 h WQ	4%/200 MPa 85%	-	-	[51]
Ti_45_Zr_5_Pd_40_Ni_2_Co_8_	arc melting 1273 K 3 h WQ	5%/200 MPa 90%	-	-	[51]
Ti_45_Zr_5_Pd_40_Ni_10_	arc melting 1273 K 3 h WQ	3.5%/200 MPa 93%	-	-	[51]
Ti_45_Zr_5_Pd_40_Co_10_	arc melting 1273 K 3 h WQ	0.2%/200 MPa 100%	-	-	[51]
Ti_45_Zr_5_Pd_20_Pt_25_Ni_5_	arc melting 1273 K 3 h WQ	1.8/200 MPa 82%	-	-	[52]
Ti_45_Zr_5_Pd_20_Pt_20_Ni_10_	arc melting 1273 K 3 h WQ	2.3%/200 MPa 95%	-	-	[52]
Ti_45_Zr_5_Pd_25_Pt_20_Au_5_	arc melting 1273 K 3 h WQ	2%/200 MPa 82%	-	-	[53]
Ti_45_Zr_5_Pd_25_Pt_20_Co_5_	arc melting 1273 K 3 h WQ	1.2%/200 MPa 60%	-	-	[53]
Ti_48.5_V_1.5_Pd_25_Ni_15_Cu_10_	arc melting ST	4.1%/300 MPa 100%	2%/100%	11.3	[54]
Ti_48.5_V_1.5_Pd_25_Ni_15_Cu_10_	arc melting ST + 573 K 1 h WQ	5.1%/400 MPa 100%	4.0%/100%	12.5	[54]
Ti_48.5_V_1.5_Pd_25_Ni_15_Cu_10_	arc melting ST + 973 K 8 h WQ	4%/400 MPa 100%	-	-	[54]
Ti_48.5_V_1.5_Pd_25_Pt_10_Ni_15_	arc melting 1473 K 2 h WQ	2.3%/200 MPa 92%	-	-	[55]
Ti_45_V_5_Pd_20_Pt_20_Ni_10_	arc melting 1473 K 2 h WQ	1.03%/200 MPa 70%	-	-	[55]
Ti_45_Zr_5_Pd_20_Pt_20_Ni_10_	arc melting 1473 K 2 h WQ	1.78%/200 MPa 96%	-	-	[55]
Ti_45_Zr_5_Pd_16.6_Pt_16.8_Ni_16.6_	arc melting 1473 K 2 h WQ	2.03%/200 MPa 98%	-	-	[55]
Ti_45_Zr_5_Pd_25_Pt_19_Co_6_	arc melting 1473 K 3 h WQ 473–673 K cycle	1.7%/100 MPa 100%	2.5% 673 K	-	[56]
Ti_45_Zr_5_Pd_25_Pt_19_Co_6_	arc melting 1473 K 3 h WQ 473–773 K cycle	2.4%/100 MPa 100%	2.7% 773 K	-	[56]

## Data Availability

No new data were created or analyzed in this study. Data sharing is not applicable to this article.

## Abbreviations

The following abbreviations are used in this manuscript:

SMA	Shape Memory Alloys
HESMA	High Entropy Shape Memory Alloy
HTSMA	High Temperature Shape Memory Alloy
HEA	High-Entropy Alloy
MEA	Medium-Entropy Alloy
MPEA	Multi-Principal Element Alloy
MT	Martensitic Transformation
MTT	Martensitic Transformation Temperature
*M*_s_	Martensite Start Temperature
*M*_f_	Martensite Finish Temperature
*A*_s_	Austenite Start Temperature
*A*_f_	Austenite Finish Temperature
*M*_p_	Peak Temperature During Martensitic Transformation
*A*_p_	Peak Temperature During Reverse Martensitic Transformation
SME	Shape Memory Effect
SE	Superelasticity
eCE	Elastocaloric Effect
C_V_, VEC	Valence Electron Concentration
ML	Machine Learning
GPM	Gaussian Process Model
DFT	Density Functional Theory
MLR	Multi-Linear Regression
RRR	Ridge Regression
SVR	Support Vector Machine Regression
KNN	K-Nearest Neighbors
LASSO	Least Absolute Shrinkage and Selection Operator
DT	Simple Decision Tree
RF	Random Forest
AdaBoost	Adaptive Boosting
XGBoost	Extreme Gradient Boosting
LCBM	Light Gradient Boosting Machine
CT	Catboost Classifier and Regressor
ANN	Artificial Neural Network Regressor
SHAP	Shapley Additive Explanations
XGBR	Extreme Gradient Boosting Regression
PIML	Physics-Informed Machine Learning
